# Increased free Zn^2+^ correlates induction of sarco(endo)plasmic reticulum stress *via* altered expression levels of Zn^2+^‐transporters in heart failure

**DOI:** 10.1111/jcmm.13480

**Published:** 2018-01-15

**Authors:** Yusuf Olgar, Aysegul Durak, Erkan Tuncay, Ceylan Verda Bitirim, Evren Ozcinar, Mustafa Bahadir Inan, Zeynep Tokcaer‐Keskin, Kamil Can Akcali, Ahmet Ruchan Akar, Belma Turan

**Affiliations:** ^1^ Department of Biophysics Ankara University Faculty of Medicine Ankara Turkey; ^2^ Department of Cardiovascular Surgery, Heart Center Ankara University Faculty of Medicine Ankara Turkey; ^3^ Department of Molecular Biology Acibadem University Istanbul Turkey

**Keywords:** zinc transporters, intracellular zinc, heart failure, endoplasmic reticulum stress, left ventricle

## Abstract

Zn^2+^‐homoeostasis including free Zn^2+^ ([Zn^2+^]_i_) is regulated through Zn^2+^‐transporters and their comprehensive understanding may be important due to their contributions to cardiac dysfunction. Herein, we aimed to examine a possible role of Zn^2+^‐transporters in the development of heart failure (HF) *via* induction of ER stress. We first showed localizations of ZIP8, ZIP14 and ZnT8 to both sarcolemma and S(E)R in ventricular cardiomyocytes (H9c2 cells) using confocal together with calculated Pearson's coefficients. The expressions of ZIP14 and ZnT8 were significantly increased with decreased ZIP8 level in HF. Moreover, [Zn^2+^]_i_ was significantly high in doxorubicin‐treated H9c2 cells compared to their controls. We found elevated levels of ER stress markers, GRP78 and CHOP/Gadd153, confirming the existence of ER stress. Furthermore, we measured markedly increased total PKC and PKCα expression and PKCα‐phosphorylation in HF. A PKC inhibition induced significant decrease in expressions of these ER stress markers compared to controls. Interestingly, direct increase in [Zn^2+^]_i_ using zinc‐ionophore induced significant increase in these markers. On the other hand, when we induced ER stress directly with tunicamycin, we could not observe any effect on expression levels of these Zn^2+^ transporters. Additionally, increased [Zn^2+^]_i_ could induce marked activation of PKCα. Moreover, we observed marked decrease in [Zn^2+^]_i_ under PKC inhibition in H9c2 cells. Overall, our present data suggest possible role of Zn^2+^ transporters on an intersection pathway with increased [Zn^2+^]_i_ and PKCα activation and induction of HF, most probably *via* development of ER stress. Therefore, our present data provide novel information how a well‐controlled [Zn^2+^]_i_ *via* Zn^2+^ transporters and PKCα can be important therapeutic approach in prevention/treatment of HF.

## Introduction

Advanced HF is an irreversible process while numerous different signalling pathways and mechanisms are involved during its development. The association between defective cardiac activity and altered cellular Ca^2+^‐homoeostasis is well characterized in HF 1. Emerging evidence suggests a central role of intracellular‐free Zn^2+^ ([Zn^2+^]_i_) in excitation–contraction coupling in cardiomyocytes by shaping Ca^2+^ dynamics 2, 3. Experimental and clinical studies have also shown that several proteins, having pivotal role in controlling cardiac contractility, are also potential targets of [Zn^2+^]_i_ as well as [Ca^2+^]_i_ 4, 5. Zinc as Zn^2+^ is required for structure and function of cells and its availability, *via* interprotein Zn^2+^‐binding sites, influences functions of numerous proteins in mammalian cells 6. Therefore, any impairment in [Zn^2+^]_i_ homoeostasis may result in a variety of cellular dysfunction including cardiomyocytes, which may in turn induce serious cardiovascular pathologies 7.

Cellular [Zn^2+^]_i_ is tightly regulated against its adverse effects through either Zn^2+^ transporters, Zn^2+^‐binding molecules or Zn^2+^ sensors, and, therefore plays a critical role in cellular signalling pathways 8, 9. In addition, it has been suggested that [Zn^2+^]_i_ associated with cellular signalling mechanisms can be classified by time windows, such as early Zn^2+^ signalling within several minutes as well as late Zn^2+^ signalling within several hours following its intracellular increase 7. However, it remains unclear whether these suggested signalling effects are independent, combinatory or cell‐dependent. Most of [Zn^2+^]_i_‐associated cellular events arised due to either the effect of mobile reactive/free Zn^2+^ but not on the non‐exchangeable Zn^2+^. Free Zn^2+^ participates in redox homoeostasis on sarco(endo)plasmic reticulum {S(E)R} function and mitochondrial metabolism, as well 10, 11, 12, 13, 14. Only few studies have documented the role of Zn^2+^ homoeostasis in cardiovascular dysfunction. These studies reported the role of Zn^2+^ dyshomeostasis in the pathogenesis of myocardial ischaemia/reperfusion injury 15, the role of a Zn^2+^ transporter, ZIP12, in the regulation of the pulmonary vascular response to chronic hypoxia 16 and the role of crosstalk between ZnT‐1 and the L‐type Ca^2+^ channels in cardiac electrical remodelling of adult rat atrium and in humans with atrial fibrillation 17, 18.

ER stress is one of the underlying mechanisms of major diseases associated with cardiac dysfunction including diabetic cardiomyopathy 10, 19. Indeed, we have previously shown that S(E)R function can be normalized when [Zn^2+^]_i_ is kept at normal level *via* enhancement of the antioxidant defence in diabetic rats 3, 11. Recently, we have shown that Zn^2+^ decrease in S(E)R leads to the up‐regulation of ER stress confirming the requirement of Zn^2+^ for proper S(E)R function 20. However, there are no clear data on the role of Zn^2+^ transporters controlling [Zn^2+^]_i_ in cardiomyocytes during the progression of HF, and, further studies are needed to clarify this important possible relation.

Excess [Zn^2+^]_i_ could affect the function of the ZIP and ZnT families, including the enhancements of protein kinase C (PKC) signalling and activation in Akt and ERK pathways 21. Particularly, free [Zn^2+^]_i_ can increase the activity of PKC by contributing to its binding to plasma membrane and hence play a crucial role in many signal transduction pathways 21, 22, 23. Furthermore, one isoform of PKC, PKCα, has been shown to have unique properties among other PKC isotypes in terms of induction of cardiac hypertrophy, and regulation of contractility and HF susceptibility 24, 25, 26. Moreover, we recently have shown that hyperglycaemia induced marked changes in ZIP7 and ZnT7 expression levels underlined excess Zn^2+^ release from S(E)R and could mediate ER stress in the heart 20.

Therefore, in this study, we first aimed to test the possible roles of some Zn^2+^ transporters in the development of HF *via* induction of ER stress. For this purpose, we, for the first time, monitored subcellular localizations of Zn^2+^ transporters such as ZIP8, ZIP14 and ZnT8 in cardiomyocytes. We also examined the role of [Zn^2+^]_i_ in HF‐modelled cardiomyocytes with doxorubicin‐treated cells (embryonic left ventricular rat heart cell line, H9c2 cells) and protein expressions levels of these Zn^2+^ transporters in human HF samples (end‐stage failing hearts, due to dilated or ischaemic cardiomyopathy) and in HF‐modelled cells. Lastly, we monitored the onset of ER stress using its markers such as GRP78, CHOP/Gadd153 and calnexin as well as phosphorylation level of PKCα in heart preparations or in directly [Zn^2+^]_i_ increased cardiomyocytes.

## Materials and methods

### Patients and tissues

Patients, who were scheduled to undergo orthotopic heart transplantation for end‐stage HF at the Department of Cardiovascular Surgery of Ankara University Faculty of Medicine, were eligible to participate in the study. The Local Ethics Committee of Ankara University approved the study protocol (1003201404‐142‐14). The investigation conforms to the principles outlined in the Declaration of Helsinki. All participating patients signed informed written consent before surgery. Left ventricular tissues were collected from discarded hearts of advanced HF patients. The cause of HF was dilated cardiomyopathy (DCMP) in two patients, and one patient had ischaemic cardiomyopathy (ICMP). Control left ventricular tissues were obtained from deceased donors that were unsuitable for cardiac transplantation. Donor families were also consented to use of the cardiac tissues for research. Clinical characteristics of patients and controls are given in Table 1.

**Table 1:** Characteristics and haemodynamic performance of patients and controls

Gender	Age (years)	LVEF (%)	PCWP (mmHg)	PVR (Wood)	CI (l/min/m^2^)	PA (mmHg)	Diagnosis
Male	46	65	11	1.6	3.8	25	DD
Male	57	60	13	2.6	2.9	35	DD
Male	31	10	33	3.6	1.52	62	DCMP
Male	46	15	11	2.0	2.5	24	ICMP
Male	48	15	18	2.9	1.27	35	DCMP

LVEF, left ventricular ejection fraction; PCWP, pulmonary capillary wedge pressure; PVR, pulmonary vascular resistance; CI, cardiac index; PA, pulmonary arterial pressure; DD, deceased donor; DCMP, dilated cardiomyopathy; ICMP, ischaemic cardiomyopathy.

### Cell line and treatment of cells with doxorubicin

We used cardiac myoblasts from left ventricle of the embryonic rat heart (ATCC CRL1446; purchased from American Type Culture Collection, Manassas, VA). Cells were grown at a density of about 10^5^ cells/cm^2^ in DMEM modified using 5.5 mM glucose instead of 25 mM and supplemented with 10% foetal calf serum, 50 U/ml penicillin‐G and 50 μg/ml streptomycin in a humidified atmosphere of 95% air and 5% CO_2_ at 37^ο^C.

To model HF in cardiomyocytes, the procedure as described previously was performed 27. The cultured H9c2 cardiomyocytes in media containing 0.5% bovine serum albumin were treated with a doxorubicin analog agent adriamycin (1 μM for 24 hrs) at 37°C. Cell viability and apoptosis were evaluated by MTT cell viability assay and some apoptosis‐related signal proteins.

### Localization examination of Zn^2+^ transporters

Localizations of Zn^2+^ transporters, ZIP8, ZIP14 and ZnT8 in cardiomyocytes were determined using anti‐ZIP8 (ProteinTech, Rosemont, IL, USA, 20459‐1; 1:50), anti‐ZIP14 (ThermoFisher, Waltham, MA, USA, PA5‐21077; 1:50) and anti‐ZnT8 (SantaCruz, Dallas, Texas, USA, sc98243; 1:50) antibodies, using confocal microscopy (Zeiss LSM 700). The S(E)R localization was determined by transfection of H9c2 cells with plasmids encoding ER‐resident Discosoma red fluorescent protein (dsRED‐ER(red) as a manner of 2 μg per well in a 6‐well plate) using Lipofectamine 2000 for 24 hr. After fixation and permeabilization of H9c2 cells with 4% paraformaldehyde and 0.3% Triton‐X100, respectively, they were incubated with ZIP8, ZIP14 and ZnT8 antibody to monitor the localization of ZIP8, ZIP14 and ZnT8 protein in the S(E)R. After overnight incubation of the cells, they were further incubated with appropriate secondary antibodies in the presence of 5% (w/v) BSA (Alexa Fluor 488 Donkey anti‐Rabbit for ZIP8 and ZnT8 (green); 1:1000, Alexa Fluor 488 Rabbit anti‐Goat for ZIP14 (green); 1:1000).

Plasma membrane was labelled using a plasma membrane marker anti‐plasma membrane Ca^2+^‐ATPase (PMCA) monoclonal antibody (ThermoFisher; MA3‐914; 1:100). After fixation and permeabilization, H9c2 cells were incubated plasma membrane marker PMCA and ZIP8, ZIP14 or ZnT8 antibody separately to demonstrate the localization of the transporters on plasma membrane. Following overnight incubation, cells were further incubated with appropriate secondary antibodies (Alexa Fluor 488 Donkey anti‐Rabbit for ZIP8 and ZnT8 (green); 1:1000, Alexa Fluor 488 Rabbit anti‐Goat for ZIP14 (green); 1:1000 and Alexa Fluor 555 Goat anti‐Mouse for PMCA (red); 1:1000) and were mounted with medium containing DAPI (blue). Images for co‐localization were analysed and processed using JACOP imageJ plugin.

### Intracellular‐free Zn^2+^ measurement in H9c2 cells

To monitor the basal (resting) level of intracellular‐free Zn^2+^ ([Zn^2+^]_i_) in H9c2 cells, we used a Zn^2+^ sensitive fluorescence dye‐loaded cells, using non‐ratiometric FluoZin‐3 (3‐μM FluoZin‐3 AM) for confocal microscope (LEICA SP5). Florescence intensities were acquired at 1 Hz, 490 nm excitation wavelength and collected at 525 nm. Image analysis was performed with ImageJ software. The steady state fluorescence intensity (*F*) was measured, then maximum and minimum ratios were determined to calculate free Zn^2+^ level using the following formula: [Zn^2+^] = *K*_d_(*F*−*F*_min_)/(*F*_max_−*F*), where the *K*_d_ for FluoZin‐3 is 15 nM. The maximum fluorescence (*F*_max_) was obtained upon Zn^2+^ saturation with Zn^2+^ salt of 1‐hydroxypyridine‐2‐thione, Zn^2+^‐pyrithione (Zn^2+^/Pyr; 10 μM), and the minimum ratio (*R*_min_) was obtained upon intracellular Zn^2+^ chelation with N, N, N', N'‐tetrakis(2‐pyridylmethyl)ethylenediamine (TPEN; 50 μM).

### Western blot analysis

Following pulverization of the samples at liquid nitrogen and homogenization, the lysates and tissues homogenates were extracted with NP‐40 lysis buffer (250 mM NaCl, 1% NP‐40 and 50 mM Tris‐HCl; pH 8.0 and 1XPIC). The protein concentrations of supernatants after centrifugation (12,000 × g, 5 min. at 4°C) were measured with the BCA assay kit (Thermo Scientific Pierce, Waltham, MA USA) according to manufacturer's instructions. Equal protein amounts were separated on 12% SDS‐PAGE Tris‐glycine or 4–12% Bis‐Tris gels (Thermo Scientific, Life Technologies, Waltham, MA, USA), as appropriate. Proteins were transferred to PVDF membranes and blocked with 4% BSA in PBS‐Tween. Membranes were probed overnight with primary antibodies diluted in 4% BSA in PBS‐Tween. The membranes were probed with antibodies against ZIP8 (Protein Tech, 20459‐1‐AP; 1:300), ZnT8 (Santa Cruz, Sc98243; 1:100), ZIP14 (Thermo, PA5‐21077; 1:300), GRP78 (Santa Cruz, Sc13968; 1:200), CHOP/Gadd153 (Santa Cruz, Sc7351, 1:500), Calnexin (Santa Cruz, Sc11397; 1:300), total PKC (Santa Cruz, Sc80; 1:250), PKCα (Santa Cruz, Sc208; 1:100), phospho‐PKCα (Santa Cruz, Sc12356; 1:100), CK2 (α1/2; Santa Cruz, Sc12738; 1:300), PUMA (α/β, Santa Cruz, Sc28226; 1:250), α‐actinin (PA517308, Thermo Fisher Sci.), PKC inhibitor (GF109203X hydrochloride, Sigma‐Aldrich), GAPDH (Santa Cruz, Sc365062; 1:1000) and β‐actin (Santa Cruz, Sc47778; 1:500) in BSA/PBS/Tween‐20 solution. Specific bands were visualized with HRP‐conjugated compatible secondary antibodies (anti‐mouse: 1:7500, anti‐goat: 1:7500, anti‐rabbit: 1:2000) and detected by ImmunoCruz Western Blotting Luminol Reagent (Santa Cruz, Sc2048). The density of bands was analysed using ImageJ software.

### Statistical analysis

Continuous data were expressed as mean (±S.E.M.), and categorical data as percentages. Groups were tested and compared using one‐way anova and Tukey post hoc test. A value of *P* ≤ 0.05 was considered statistically significant. For all data, no statistics were used for predetermination of the sample size, randomization or blinding.

## Results

### Outcome of patients

We used left ventricular samples from three patients and two controls. Three patients underwent orthotopic heart transplantation with end‐stage HF. One of these patients had ICMP while the other two had DCMP (HF group). We obtained control group samples from deceased donors that were unsuitable for cardiac transplantation (DD group). Both controls have good left ventricular function. One control deceased donor had Hepatitis C, and the other heart of the deceased donor was discarded because of persistant hypovolemic shock due to multiple traumatic injuries. The haemodynamic characteristics of patients and controls are summarized in Table 1. As mentioned previously, an important biochemical marker in heart failure is B‐type natriuretic peptide (BNP), which is produced in heart ventricles in response to increased mechanical load and wall stretch 28. Therefore, before undergoing orthotopic heart transplantation with end‐stage HF, the plasma BNP levels were measured in patients. The BNP level was in the range of 3000–4000 pg/ml in group of HF while this value was 2000–2400 pg/ml in DD group. The difference between these two groups is significantly different from each other (*P* < 0.001).

### Demonstration of subcellular localizations of ZIP8, ZIP14 and ZnT8 in cardiomyocytes

**Figure 1:**
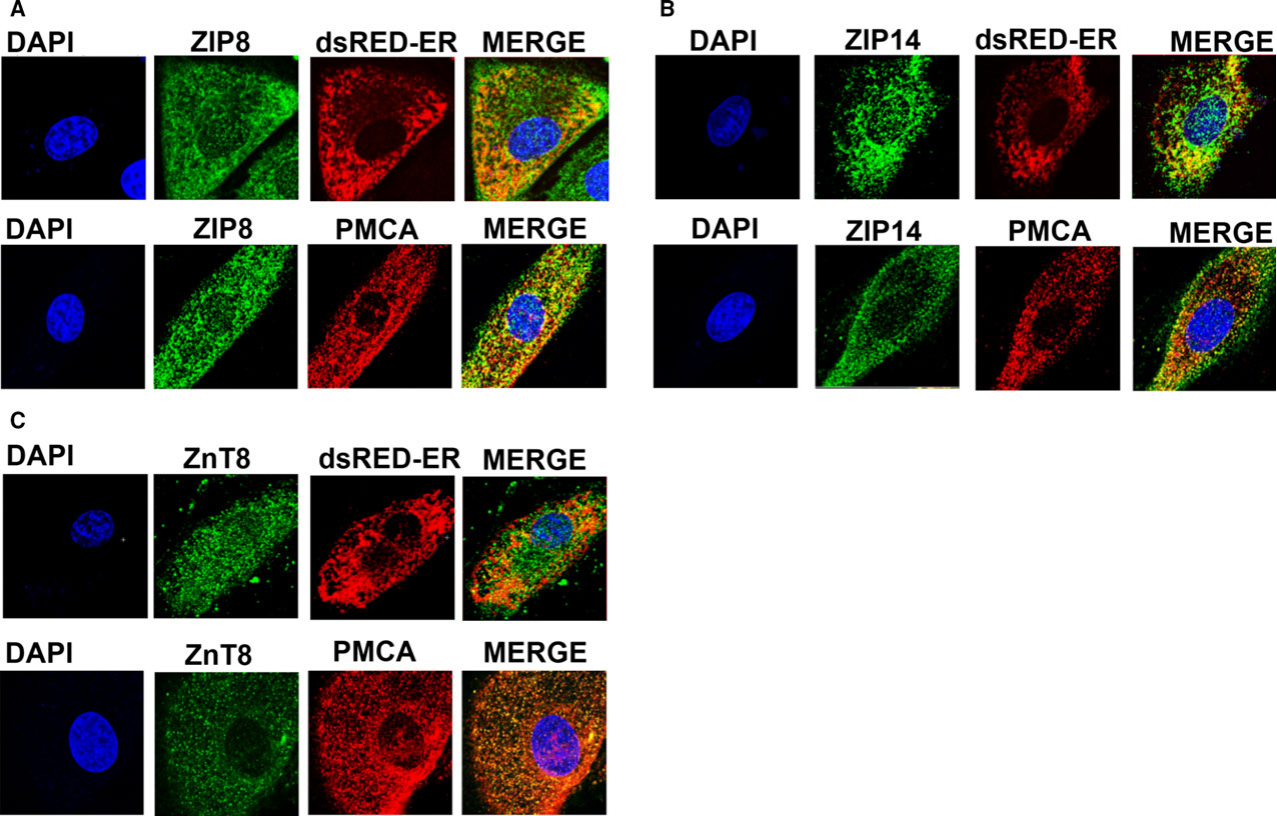
Visualization of ZIP8, ZIP14 and ZnT8 localizations in H9c2 cells. Localizations either to the S(E)R or to the sarcolemma/plasma membrane of ZIP8 (**A**), ZIP14 (**B**) and ZnT8 (**C**) were visualized using confocal micrographs in H9c2 cells. To examine the subcellular localizations of these transporters to S(E)R, H9c2 cells were transfected with ER‐resident discosoma red fluorescent protein (dsRed‐ER) to label ER or incubated with PMCA antibody to label sarcolemma/plasma membrane and with DAPI (blue) as well. Cells were incubated only with secondary antibodies and mounted with mounting medium contains DAPI (blue), and then images were merged.

To test whether ZIP8, ZIP14 and ZnT8 are localized to either on S(E)R, sarcolemma or both, the cells were co‐incubated with plasma membrane marker antibody (PMCA) and ZIP8, ZIP14 or ZnT8 primary antibodies after fixation and permeabilization. Images were captured using confocal microscopy and then merged (Fig. 1A–F). The Pearson's coefficients (PC) were calculated using Huygens programme for co‐localization values of on either S(E)R or sarcolemma ZIP8 (A and B), ZIP14 (C and D) and ZnT8 (E and F). The calculated PCs from images for ZIP8‐S(E)R and ZIP8‐PMCA are 0.44 ± 0.03 and 0.60 ± 0.02, while these values for ZIP14 and ZnT8 are 0.50 ± 0.08 *versus* 0.66 ± 0.03 and 0.42 ± 0.05 *versus* 0.80 ± 0.02.

Our PCs' values strongly support the localization of these three Zn^2+^ transporters on sarcolemma as about over 60% in mammalian ventricular cardiomyocytes. Additionally, our data demonstrated that these transporters were also localized on the S(E)R of the cardiomyocytes as about 40–50% due to calculated PCs' values.

### Altered expression levels of the Zn^2+^ transporters in heart failure

**Figure 2:**
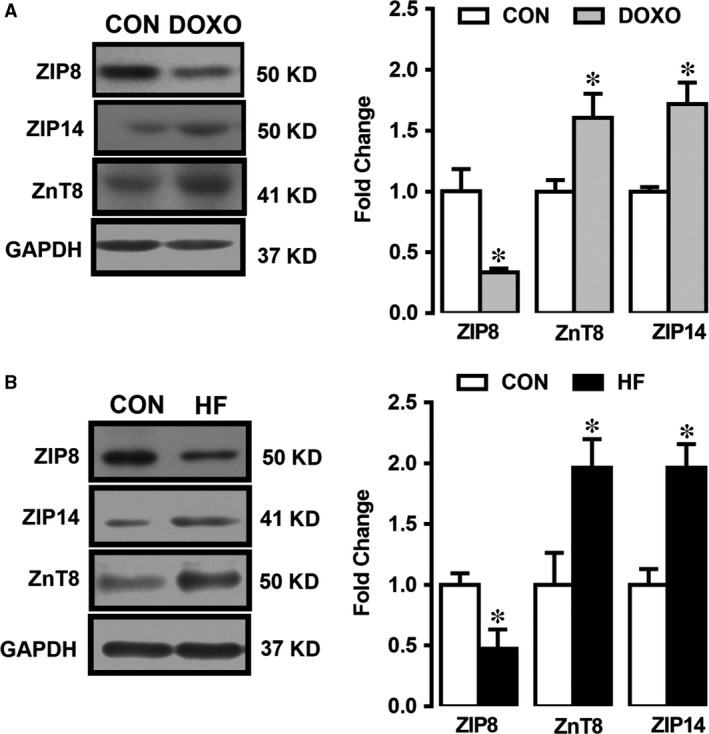
Expression levels of ZIP8, ZIP14 and ZnT8 in heart tissue homogenates and doxorubicin‐treated H9c2 cells. Representative Western blotting bands for protein expression levels are given left part of figures in (**A**) and (**B**). The densitometry analysis showing the proteins of ZIP8, ZnT8 and ZIP14 is expressed at 50 kD, 41 kD and 50 kD, respectively, in doxorubicin‐treated H9c2 cells (DOXO group) with respect to untreated cells (CON group) (**A**) and underwent orthotopic heart transplantation with end‐stage heart failure (HF group) with respect to unsuitable for cardiac transplantation patients (CON group) (**B**) GAPDH at 37 kD as reference protein. Bars represent mean (±S.E.M.) for each group. Number of human samples, *n* = 3 for HF group and *n* = 2 for CON group. All measurements with double assays in each sample from each group for each type of measurement. Significance level accepted at *P < 0.05 *versus* CON.

We examined first the expression levels of Zn^2+^ transporters ZIP8, ZIP14 and ZnT8 in human heart tissue. Western blot analysis showed that the expression levels of ZIP14 and ZnT8 were significantly higher than those of controls, whereas ZIP8 level was significantly lower comparison to the control (Fig. 2A, right) while the representative Western blot images are given also in the same figure, as well (Fig. 2A, left).

To validate the human heart data related with changes in expression levels of ZIP14, ZIP8 and ZnT8, we also examined the expression levels of these transporters in doxorubicin‐treated rat ventricular cells. The observed changes in these transporters were similar to the human data (HF). The expression levels of ZIP14 and ZnT8 were also significantly higher than those of controls while the ZIP8 level was markedly low (Fig. 2B, right). Representative Western blot images are given in the left part of Figure 2B.

### Validation of heart failure in human heart tissue and doxorubicin‐treated cells

**Figure 3:**
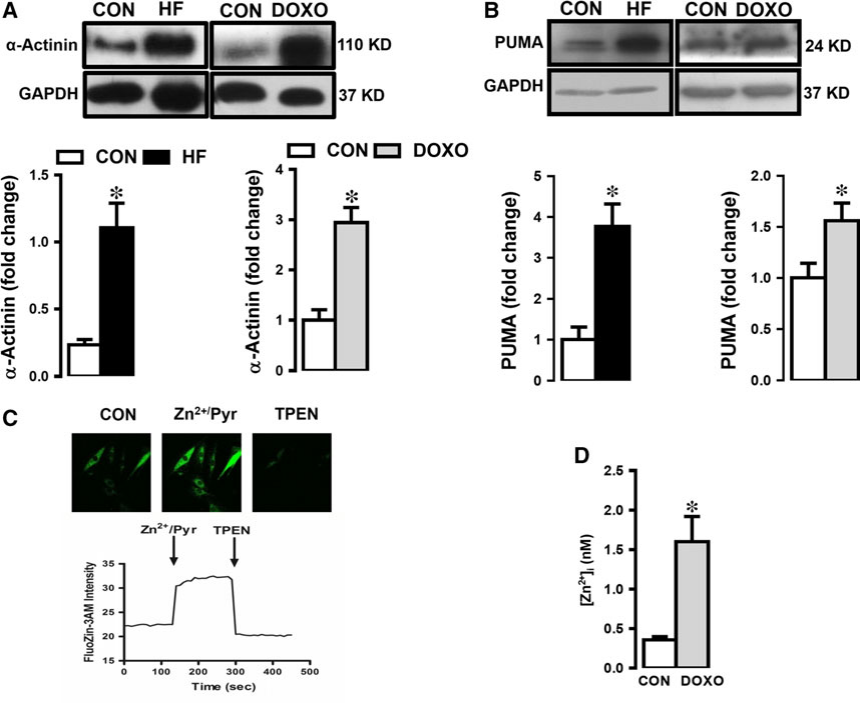
Validation of HF in human heart tissue and doxorubicin‐treated cells and demonstration of intracellular‐free Zn^2+^ increase under HF. (**A**) Expression level of α‐actinin, ACTN and PUMA in both human heart tissue with heart failure (HF) (**A**) and doxorubicin (DOXO)‐treated H9c2 cells (**B**) with respect to their controls (CON). Representative Western blotting bands for protein expression levels are given upper parts of figures in (**A**) and (**B**). (**C**) Representing of measurement protocol of intracellular‐free Zn^2+^, [Zn^2+^]_i_ in doxorubicin‐treated (failing heart modelled, DOXO group) cells, loaded with Zn^2+^ selective fluorescent dye FluoZin‐3. Zn^2+^ ionophore pyrithione, Zn^2+^/Pyr (1‐μM) exposure (3 min.) and high‐affinity heavy‐metal Zn^2+^‐chelator, N,N,N',N'‐tetrakis(2‐pyridylmethyl)ethane‐1,2‐diamine, TPEN (50 μM). The mean (±S.E.M.) intracellular Zn^2+^level, as FluoZin‐3 intensity, respectively. (**D**) Bar graphs representing the [Zn^2+^]_i_ levels calculated as nM (see Methods and materials) in the treated cells comparison with the untreated cells (CON group). Bars represent mean (±S.E.M.) for each group. Number of human samples, *n* = 3 for HF group and *n* = 2 for CON group. All measurements with double assays in each sample from each group for each type of measurement. Significance level accepted at *P < 0.05 *versus*. CON.

To correlate diastolic and systolic dysfunction in human HF and deposition of non‐sarcomeric alpha (α)‐actinin (ACTN) in HF and HF‐modelled cells, we first measured the ACTN level in underwent orthotopic heart transplantation with end‐stage HF with respect to unsuitable for cardiac transplantation patients (CON group). As can be seen from Figure 3A, expression level of ACTN in human tissue (HF group) was significantly high with respect to CON group (left). To confirm further the HF in heart tissues and cell line, we measured the expression level of this biomarker protein, ACTN in doxorubicin‐treated H9c2cells. As can be seen in Figure 3A, ACTN level was markedly high in the treated group compared to the untreated group (right).

We also examined the expression level of PUMA (as a pro‐apoptotic member of the Bcl‐2 family) to further confirmation of heart failure in both human tissues and doxorubicin‐treated H9c2 cells. As can be seen in Figure 3B, PUMA protein levels (with respect to GAPDH) were increased markedly in HF group and HF‐modelled H9c2 cells comparison with those of their controls (left and right, respectively).

### [Zn^2+^]_i_ increases in doxorubicin‐treated cardiomyocytes

To determine [Zn^2+^]_i_ in HF‐modelled cells, we measured the [Zn^2+^]_i_ changes in H9c2 cells loaded with a Zn^2+^ selective and highly sensitive fluorescent dye FluoZin‐3. Representative fluorescence intensity changes of FluoZin‐3 and experimental protocol are given in Figure 3C. Our data showed that Zn^2+^‐ionophore pyrithione, Zn^2+^/Pyr (1 μM) exposure (3 min.) induced significant increase in FluoZin‐3 intensity. Further addition of high‐affinity heavy‐metal Zn^2+^ chelator, N,N,N',N'‐tetrakis(2‐pyridylmethyl)ethane‐1,2‐diamine, TPEN (50 μM), FluoZin‐3 intensity decreased below its initial value. Of note, in our experiments, the fluorescence loading and Zn^2+^/Pyr‐induced changes in [Zn^2+^]_i_ changes as well as [Zn^2+^]_i_ chelation of [Zn^2+^]_i_ in the cells were very sharp and confirmed to be homogenous using confocal images of the loaded cells. The differences between calculated intracellular‐free Zn^2+^ levels as nM from normal and doxorubicin‐incubated H9c2 cells are given in Figure 3D. As can be seen from the bar graphs, the [Zn^2+^]_i_ of the treated cells is about fourfold higher compared to the controls.

### ER stress sensors as molecular chaperones in human HF and doxorubicin‐treated H9c2 cells

**Figure 4:**
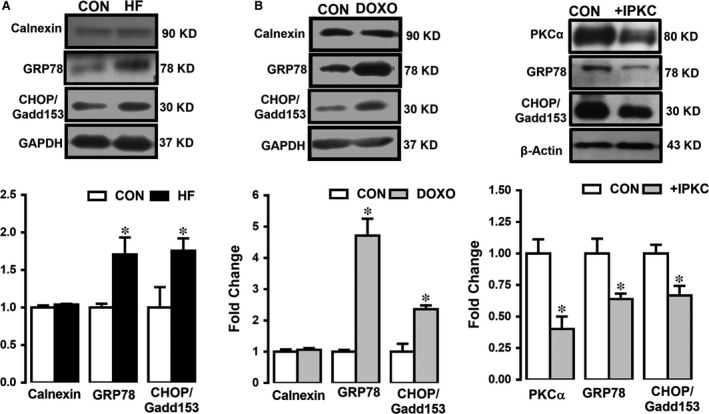
Western blot analysis showing the expression levels of ER stress/chaperone proteins in human and doxorubicin‐treated H9c2 cells. The densitometry analysis showing the protein levels of GRP78, CHOP/Gadd153 and calnexin at 94 kD, 30 kD and 90 kD, respectively, as well as PKCα activation status detected as protein expression level at 80 kD (with respect to GAPDH at 37 kD) in human heart failure (HF) (**A**) and in doxorubicin (DOXO)‐treated H9c2 cells (**B**) with respect to their controls (CON group). Representative Western blotting bands for protein expression levels are given upper parts of figures in (**A**) and (**B**). Bars represent mean (±SEM) for each group. Number of human samples, *n* = 3 for HF and *n* = 2 for CON. Significance level accepted at *P < 0.05 *versus* CON and ^#^ *P* < 0.05 *versus* TUDCA group.

As GRPs (ER‐targeted cytoprotective chaperones, the unfolded‐protein response signalling‐proteins), as molecular chaperones, are regulated by signal transduction pathways originating in the ER, we first examined the protein expression levels of GRP78, CHOP/Gadd153 and calnexin in the human tissues. Expression levels of GRP78 and CHOP/Gadd153 in human HF group were significantly increased with respect to the controls while no change in calnexin expression level (Figure 4A).

To test whether the inhibition of an endogenous kinase, protein kinase C (PKC), which is generally activated under pathological condition due to different cellular alterations including cellular Zn^2+^ level 22, 23 lead to the suppression of ER stress, we performed another group of measurements. As can be seen in Figure 4C, PKC inhibition with an inhibitor (IPKC; incubation of H9c2 cells with 0.1 μM GF109203X hydrochloride for 24 hrs) prevented the induction of ER stress, significantly.

### Verification of increased [Zn^2+^]_i_‐associated ER stress in doxorubicin‐treated H9c2 cells

**Figure 5:**
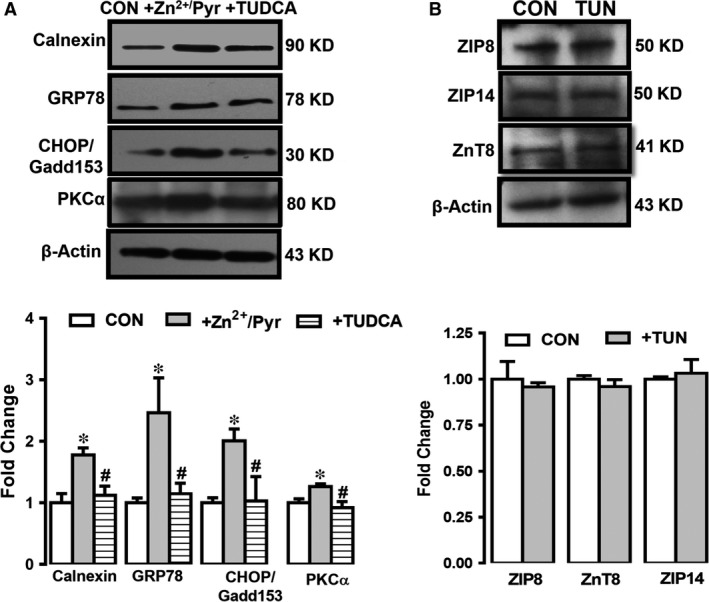
Verification of the role of altered expression levels of Zn^2+^ transporters on increased [Zn^2+^]_i_‐associated ER stress in HF. The ER stress marker proteins such as GRP78, CHOP/Gadd153 and calnexin at 94 kD, 30 kD and 90 kD (with respect to GAPDH at 37 kD) in directly [Zn^2+^]_i_ increased H9c2 cells with Zn^2+^ ionophore pyrithione, Zn^2+^/Pyr (0.1 μM for 24 hrs) incubation (without or with an ER stress inhibitor tauroursodeoxycholic acid, TUDCA, 50 μM for 18 hrs) H9c2 cells with respect to their controls (CON) (**A**). The densitometry analysis showing the protein levels of GRP78, CHOP/Gadd153 and calnexin at 94 kD, 30 kD and 90 kD, respectively (with respect to GAPDH at 37 kD). (**B**) The expression levels of ZIP8, ZIP14 and ZnT8 in a direct ER stress activator tunicamycin incubated H9c2 cells (TUN; 10 μM for 18 hrs). All measurements with double assays in each sample from each group for each type of measurement. Significance level accepted at *P < 0.05 *versus* CON.

To verify whether increased [Zn^2+^]_i_ underlies the induction of ER stress in HF‐modelled H9c2 cells, we first incubated H9c2 cells with Zn^2+^‐ionophore pyrithione, Zn^2+^/Pyr (0.1 μM for 24 hrs) and then measured the levels of ER stress chaperones mentioned the previous section. As can be seen from Figure 5A, the expression levels of GRP78, CHOP/Gadd153 and calnexin increased significantly when [Zn^2+^]_i_ are increased.

For further confirmation of this experimental approach, in another set of experiments, we measured the expression levels of these markers in the presence of ER stress inhibitor tauroursodeoxycholic acid (TUDCA; 50 μM for 18 hrs) in Zn^2+^/Pyr pre‐treated cardiomyocytes. As can be seen from Figure 5A (last columns), the expression of these chaperone proteins was not different from those of controls. These data can suggest a possible association between ER stress induction and increased [Zn^2+^]_i_ in human HF.

To understand the involvement of PKCα either under increased [Zn^2+^]_i_ or with TUDCA which directly inhibits ER stress, we measured PKCα expression level in these groups. As can be seen in Figure 5A (last column), PKCα expression level was increased significantly under increased [Zn^2+^]_i_ with respect to the controls while its level was not changed in ER stress inhibited cells.

### The [Zn^2+^]_i_ increase but not a direct ER stress induction underlies the changes in the altered expression levels of Zn^2+^ transporters in HF‐modelled doxorubicin‐treated H9c2 cells

To verify whether ER stress response activation accounts for cardiomyocyte dysfunction obtained in doxorubicin‐incubated H9c2 cells, we examined the expression levels of these chaperones in a direct ER stress activator tunicamycin incubated H9c2 cells (TUN; 10 μM for 18 hrs). As seen in Figure 5B, the expression levels of none of these Zn^2+^ transporters were not changed, significantly. From these data, one can provide a hypothesis that ER stress due to any sources cannot directly induce changes in the expression levels of ZIP8, ZnT8 and ZIP14 except increased [Zn^2+^]_i_. Therefore, one can further hypothetize that ER stress can be the end process following the changes in these transporters, most probably due to increased [Zn^2+^]_i_ in mammalian HF condition.

### The endogenous kinases in failing human heart

**Figure 6:**
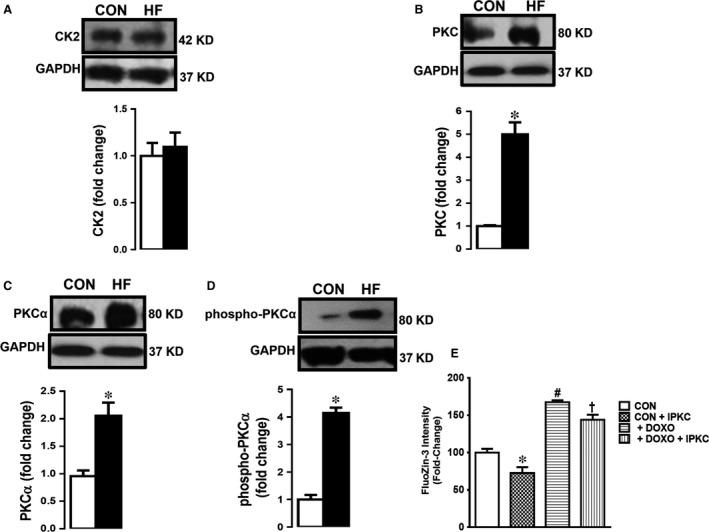
Endogenous kinases CK2 and PKC in the human heart tissues. The densitometry analysis of the protein expression levels (Western blotting) given as original bands and mean (±S.E.M.) values for CK2 (α1/α2) in (**A**), total PKC in (**B**), phospho‐PKCα (right) and PKCα (left) in (**C**) with respect to GAPDH at 37 kD given for human (HF) and group comparison with the controls. The data were obtained after duplicate assays of each sample from each group for each type of measurement. The number of samples used are the same given in Figure 4 for groups. (**D**) The [Zn^2+^]_i_ levels measured in doxorubicin‐treated cells with and without a total PKC inhibitor (IPKC GF109203X; 0.1 μM for 24‐hrs incubation). Bars represent mean (±S.E.M.). Significance level accepted at *P < 0.05 *versus* CON group, ^#^ *P* < 0.05 *versus* CON group and ^†^ *P* < 0.05 *versus* DOXO group.

To extend our testing on possible association between HF, increased [Zn^2+^]_i_ and role of protein kinase‐2 (CK2) on triggering Zn^2+^‐signalling pathways by phosphorylating Zn^2+^ transporters 29 and contributing to Zn^2+^ homoeostasis, particularly under pathological conditions 20, 30, we measured the expression level of CK2α. As can be seen from Figure 6A, the CK2α level in human HF was not significantly different from that of controls.

As a PKC isoform, PKCα, has been shown to have unique properties among the PKC isotypes such as induction of cardiac hypertrophy 25, regulation of contractility and heart failure susceptibility 26, we aimed to examine this parameter in human HF samples.

The expression level of PKCα was markedly high in human HF group compared to the CON group (Fig. 6C). Furthermore, we also determined the phosphorylation level of PKCα (phospho‐PKCα) in the same samples. As can be seen in Figure 6D, the expression level of phospho‐PKCα was significantly increased in HF group comparison with the CON group.

### Validation of the role of PKCα‐phosphorylation under increased [Zn^2+^]_i_ in human HF

To test further the role of PKCα‐phosphorylation in [Zn^2+^]_i_ increase in mammalian HF, we measured [Zn^2+^]_i_ level in both control and doxorubicin‐treated cells with and without a total PKC inhibitor (IPKC; 0.1 μM for 24 hrs incubation). As shown in Figure 6E, the increased [Zn^2+^]_i_ level in doxorubicin‐treated H9c2 cells was significantly decreased while there was also decrease in the untreated cells.

These data are prominent confirmation of association between increased [Zn^2+^]_i_ and phosphorylation of PKCα in mammalian HF.

## Discussion

In the present study, we provide evidence to support the crucial role of increased [Zn^2+^]_i_ on mammalian heart dysfunction *via* alterations of protein expression levels of Zn^2+^ transporters and induction of ER stress, most probably due to phosphorylation of PKCα. Additionally, our present data further suggest that expression level of some Zn^2+^ transporters together with PKCα activation may play important role in human HF *via* induction of ER stress. Therefore, our observation related with the activation of PKCα under an increased [Zn^2+^]_i_ but no change in ER stress markers under PKC inhibition as well as no increase in [Zn^2+^]_i_ of the doxorubicin‐treated cells under PKC inhibition suggests the possible role of these Zn^2+^–transporters. These information led us to have a conclusion of increase [Zn^2+^]_i_ and PKCα‐activation (on a way of intersection‐pathway associated with them) induced HF, *via* induction of ER stress. Therefore, they have a paramount importance since [Zn^2+^]_i_ *via* Zn^2+^ transporters and PKCα may be novel therapeutic approach in prevention/treatment of human HF. Zinc, particularly as Zn^2+^, is essential for human health, and disturbances in its homoeostasis can contribute and/or exacerbate the pathology observed in many chronic conditions including cardiovascular diseases 31, 32, 33. Although Zn^2+^ is required by all cell types, and its toxicity is relatively rare, [Zn^2+^]_i_ is tightly controlled under physiological conditions by several ways including specific Zn^2+^ transporters, at most, in redox homoeostasis of the cells 34. Yet, the overall physiological importance of [Zn^2+^]_i_ and Zn^2+^‐transporters at the whole‐organism level, particularly their interaction with pathological conditions, remains unclear. We examined possible roles of the Zn^2+^ transporters ZIP8, ZIP14 and ZnT8 in cardiomyocytes under failing condition. For this essential aim, we first examined their protein levels in failing heart preparations. Under any types of heart failure, cardiomyocytes significantly decreased protein level of ZIP8 with markedly increased level of ZIP14 and ZnT8. Additionally, high [Zn^2+^]_i_ existence of ER stress together with marked apoptotic status validated the failing heart in our samples. We also, for the first time, presented the subcellular localizations of these transporters in cardiomyocytes. Here, we demonstrated that ZIP8, ZIP14 and ZnT8 localized to both S(E)R and sarcolemma in H9c2 cells, further indicating multiple localization sides for these Zn^2+^ transporter in cardiomyocytes. Taken into consideration a possible crosstalk between increased [Zn^2+^]_i_, induction of ER stress and apoptosis in cardiomyocytes, although not assessed directly, it is very logical to hypothesize that any change in any Zn^2+^ transporter under pathological conditions is likely to further exacerbate these changes and contributes to the deleterious consequences of Zn^2+^ redistribution between compartments. Importantly, these transporters are shown to localize into S(E)R membrane and may thus operate as a functional couple catalysing Zn^2+^ release and uptake respectively from the S(E)R while S(E)R is a very good candidate as Zn^2+^ pool 2, 5, 20.

Herein, we have shown a close correlation between Zn^2+^ transporters, [Zn^2+^]_i_ and ER stress in failing heart. Our present data demonstrated that ER stress is not induced directly but, our data showed that increased [Zn^2+^]_i_ due to alterations in the Zn^2+^ transporters induces ER stress chaperons. ER stress is associated with a range of diseases, including ischaemia/ reperfusion injury and other heart diseases, making ER stress a probable instigator of pathological cell death and dysfunction 35. The role of ER stress in heart disease has not been extensively studied, but it is well accepted that disturbances in the normal functions of the ER lead to an evolutionarily conserved cell stress response, which is aimed initially at compensating for damage but can eventually trigger cell death if ER dysfunction is severe or prolonged. The principal challenge with any strategy for blocking cell death caused by ER stress lies with the multitude of parallel pathways potentially leading to downstream cell death mechanisms. Thus, blocking only one cell death pathway emanating from the ER may be inadequate to preserve cell survival. Further studies of genes and gene products involved in ER stress‐initiated cell death are needed to fully validate targets for drug discovery.

In the present study, we also confirmed the development of HF in human tissue and HF‐modelled cardiomyocytes by measuring high level of non‐sarcomeric alpha (α)‐actinin (ACTN). Indeed, correlation between morphological changes, including development of fibrosis, and diastolic and systolic dysfunction together with deposition of ACTN in cardiomyocytes from patients with dilated cardiomyopathy or chronic pressure overload has been shown, previously 36, 37. Although the precise mechanism of ER stress‐induced cardiomyocyte apoptosis remains elusive; it is believed that the mitochondrial apoptotic machinery is recruited through up‐regulation of PUMA, a pro‐apoptotic member of the Bcl‐2 family. Importantly, we and others have shown that any suppression of PUMA activity could prevent both ER stress and ischaemia/reperfusion‐induced or diabetes‐induced cardiomyocyte loss 38. In parallel to these data, we also found PUMA levels were increased markedly in all failing heart models compared to the controls suggesting a signalling mechanism related with ER stress‐mediated apoptosis.

Although there are several studies related with ZIP8, ZIP14 and ZnT8, there are missing data associated with these transporters and cardiovascular complications. As summary of role of ZIP8 in mammalian cells, Galvez‐Peralta *et al*. have shown a critical role of ZIP8‐mediated Zn^2+^ transport during in utero and neonatal growth, organ morphogenesis and hematopoiesis while its critical importance was demonstrated for Zn^2+^ cytoprotection in lung epithelia 39. ZIP14 associated studies pointed out the expression of this transporter in heart tissue besides the other organs in mammalians. Previous studies have shown that ZIP14 is localized at the plasma membrane and in transferrin‐containing endosomal compartments 40, 41. Recent studies demonstrated that the level of ZIP14 protein is increased in the liver of rats fed a high‐iron diet and in iron‐loaded human hepatoma cells, suggesting that ZIP14 contributes to tissue iron loading under high‐iron conditions 41. A tissue expression array shows that ZIP14 mRNA is ubiquitously expressed with high levels in the liver, pancreas and heart 40.

The ZnT8 is exclusively expressed in pancreatic β‐cells and encodes a protein that transports Zn^2+^ from cytoplasm to insulin secretory vesicles 42. Additionally, genomewide‐associated studies and recent meta‐analysis studies demonstrated that a polymorphism in ZnT8 has closely associated with increased risk of impaired glucose regulation and type 2 diabetes 12, 43, 44. In studies on multiple Zn^2+^ transporters, such as ZIP8 and ZIP14 or ZIP14 and ZnT10, it has been mentioned that they can be functioning together and thus enhancing their roles in cellular signalling mechanisms 45.

It is well documented that several endogenous substrate proteins of mammalian heart can be phosphorylated with PKC isozymes while they can be activated under pathological conditions including high oxidative stress status *via* thiol oxidation and release of Zn^2+^ from cysteine‐rich region of PKC 46, 47. In here, we have shown that PKCα can be activated under increased [Zn^2+^]_i_ in cardiomyocytes while its activation can be prevented by ER stress inhibition. Additionally, we have also shown that if we inhibited PKCα, then ER stress can also be inhibited. Therefore, one can suggest that ER stress can be prevented if we have a well‐controlled [Zn^2+^]_i_ *via* inhibition of PKCα even if under HF. Indeed, studies pointed out the important role of activated PKCα in the heart, as an important mediator of induction of ventricular arrhythmias 48. It has also been shown that PKCα along with [Zn^2+^]_i_ and Zn^2+^ transporters function as effectors of oxidative tissue injury, in part, *via* induction of ER stress 49, 50. Particularly, PKCα is a specific member of the protein kinase family and is unique in its mode of regulation compared to other kinases within this family. However, it seems CK2 does not underlie any Zn^2+^‐related mechanisms in mammalian heart failure, although it has important role in function of cancer cells *via* ZIP7 40.

**Figure 7:**
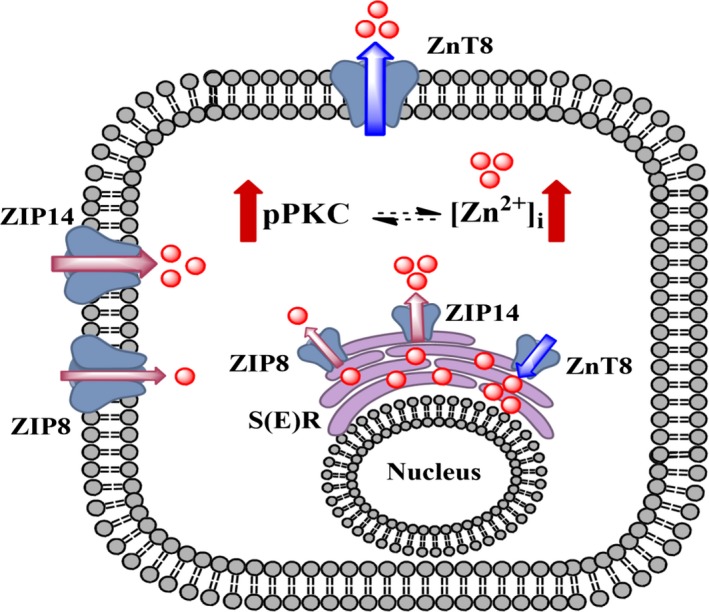
A putative summarized presentation of our data on a possible role of Zn^2+^ transporters on an intersection‐pathway associated with increased cytosolic free Zn^2+^ and PKCα‐activation and induction of heart failure *via* development of ER stress. The thickness of the arrows related with the transporters show the alterations in their expression levels. Here, thick red arrows represent increased level of pPKC as phosphorylated PKCα and increased [Zn^2+^]_i_, intracellular‐free Zn^2+^ concentration in cells under heart failure. The thickness of red arrows related with localization of Zn^2+^ transporters represent increase/decrease expression levels. S(E)R represents sarco(endo)plasmic reticulum.

A schematic presentation of our present data, given in Figure 7, shows the subcellular distribution of Zn^2+^ transporters, ZIP8, ZIP14 and ZnT8 in ventricular cardiomyocytes. Three of them are localized to both S(E) and sarcolemma and seem to be responsible for the regulation of cellular distribution of free Zn^2+^ under physiological condition. When cardiomyocytes are under pathological condition, leading to heart failure, the expression levels of these three transporters are affected through most probably phosphorylation/activation of PKCα, consequently, [Zn^2+^]_i_ is increased in cardiomyocytes. In this regard, it has been shown that PKC has ability to regulate many cardiovascular functions *via* targeting many cardiovasotropic growth factors 51, whereas [Zn^2+^]_i_ increases the interaction between PKC and actin flaments 23, 52. Therefore, PKC isoforms could be assessed as possible Zn^2+^ targets and as important regulators of cardiac function under the conditions of ischaemia reperfusion 53. The increase in [Zn^2+^]_i_ under pathological condition further underlies induction of ER stress *via* overexpression of ER stress chaperons in cardiomyocytes which is nicely confirmed with measurement of [Zn^2+^]_i_ under a PKC inhibition in doxorubicin‐treated cardiomyocytes.

Overall, our present data provide an important insight into the HF in humans, in part, due to the importance of [Zn^2+^]_i_, Zn^2+^ transporters and proper excitation–contraction coupling in cardiomyocytes, *via* an association with phosphorylation of PKCα. Indeed, Zn^2+^ is an essential cofactor for normal cell function and herein, we have shown that altered expression and function of Zn^2+^ transporters can contribute to the pathogenesis of cardiac disorders through increased ER stress and apoptosis. The expression and regulation of Zn^2+^ transporters in the heart and the toxicity of high [Zn^2+^]_i_ to these cells will open new insights into the HF in mammalians as proposing new therapeutic strategy as well as the development of Zn^2+^ containing new markers/sensors to better handle HF in humans.

### Limitations

Further studies are needed to evaluate the role of [Zn^2+^]_i_, and Zn^2+^ transporters in advanced HF patients with different aetiologies. Duration of advanced HF and medications also may affect the results.

## Ethical Approval

Patients were eligible to participate in the study and the Local Ethics Committee of Ankara University approved the study protocol (1003201404‐142‐14).

## Conflict of interest

The authors declare that there is no conflict of interest.
